# Enhancing the Uniformity of a Memristor Using a Bilayer Dielectric Structure

**DOI:** 10.3390/mi15050605

**Published:** 2024-04-30

**Authors:** Yulin Liu, Qilai Chen, Yanbo Guo, Bingjie Guo, Gang Liu, Yanchao Liu, Lei He, Yutong Li, Jingyan He, Minghua Tang

**Affiliations:** 1 School of Materials Science and Engineering, Xiangtan University, Xiangtan 411105, China; ylliuxtu@163.com; 2 Department of Micro and Nano Electronics, School of Electronic Information and Electrical Engineering, Shanghai Jiao Tong University, Shanghai 200241, China; yanbo.guo@sjtu.edu.cn (Y.G.); bingjie.guo@sjtu.edu.cn (B.G.); 3 Aerospace Science & Industry Shenzhen (Group) Co., Ltd., Shenzhen 518000, China; chenqilai8816@126.com; 4 Shi Changxu Class of the School of Materials Science and Engineering, Xiangtan University, Xiangtan 411105, China; m17382138208@163.com (Y.L.); leihe0712@163.com (L.H.); liyutong_0505@163.com (Y.L.); 202005721123@smail.xtu.edu.cn (J.H.)

**Keywords:** resistive random access memory (RRAM), crossbar array, bilayer dielectric structure, Gibbs free energy

## Abstract

Resistive random access memory (RRAM) holds great promise for in-memory computing, which is considered the most promising strategy for solving the von Neumann bottleneck. However, there are still significant problems in its application due to the non-uniform performance of RRAM devices. In this work, a bilayer dielectric layer memristor was designed based on the difference in the Gibbs free energy of the oxide. We fabricated Au/Ta_2_O_5_/HfO_2_/Ta/Pt (S3) devices with excellent uniformity. Compared with Au/HfO_2_/Pt (S1) and Au/Ta_2_O_5_/Pt (S2) devices, the S3 device has a low reset voltage fluctuation of 2.44%, and the resistive coefficients of variation are 13.12% and 3.84% in HRS and LRS, respectively, over 200 cycles. Otherwise, the bilayer device has better linearity and more conductance states in multi-state regulation. At the same time, we analyze the physical mechanism of the bilayer device and provide a physical model of ion migration. This work provides a new idea for designing and fabricating resistive devices with stable performance.

## 1. Introduction

As the limits of Moore’s Law are approached, computers using the Von Neumann architecture are limited by a storage wall and a power wall, and there is an urgent need to develop new memory-device solutions to meet the requirements of modern society for big data, artificial intelligence, and emerging industries [1,2]. Compared with the current mainstream charge-based flash memory, resistive switching random access memory (RRAM) has been considered one of the most promising prospects for next-generation non-volatile memory (NVM) devices owing to its simple structure, high integration density, high-speed operation, low power consumption, and good compatibility with conventional CMOS processes [3,4,5]. The structure of RRAM devices is similar to the traditional sandwich structure, consisting of a top electrode, a dielectric layer, and a bottom electrode. Pt, Au, Ti, Cu, Ag, or TiN are usually used as electrode materials [6,7]. Organics [8], transition metal oxides [7,9], perovskites [10], and two-dimensional materials [11] can be used as dielectric layers. Among the different materials, binary transition metal oxides are used for resistive device preparative studies owing to their simple chemical compositions [12,13], polymorphic switching properties, and compatibility with complementary metal oxide semiconductor (CMOS) fabrication processes [8].

Memory resistors can be used for storage and synapse mimicry [14,15]. Traditional methods of simulating neurons require dozens of conventional electronics, transistors, capacitors, etc. [16] This results in a huge challenge for power consumption and integration of the chip. The conductance state of the memristor is continuously adjustable under an applied electric field, but the uniformity of the memristor and the linearity of the polymorphic regulation are important performance metrics for its applicability, which has become a key parameter to be optimized [5,8,16].

Due to the existence of only a metal-semiconductor interface in the single-layer device, the concentration of oxygen ions and oxygen vacancies cannot be regulated, which increases the formation and breakage of conducting channels randomly and makes the device performance unstable [17,18]. Scientists have proposed many ways to improve stability, such as introducing nanocrystals in the functional layer [19,20,21], impurity doping [22,23], and integrating a layer of pinpoint electrodes [24,25,26]. However, these solutions require the addition of additional microstructure processing, sacrificing the scalability of micro-miniaturization and increasing production costs.

The common types of thin film growth are chemical vapor deposition, reactive sputtering, atomic layer deposition, magnetron sputtering, and sol-gel. Among these, chemical vapor deposition lacks stability in the process of growing thin films. Reactive sputtering needs to maintain a high-temperature atmosphere during growth, which makes the method incompatible with CMOS processes. Atomic layer deposition is suitable for growing uniform films on substrates with gradients, but it is costly. The sol-gel method is less costly, but its homogeneity is poor. In contrast, magnetron sputtering can grow homogeneous films in a lower-temperature atmosphere, which is favorable for film growth [27]; therefore, in this study, the magnetron sputtering technique was used to prepare dielectric films.

Therefore, we need to investigate simple and efficient methods to regulate the formation and breakage of conducting channels to improve the stability of the devices. Different Gibbs free energies lead to the varying simplicity of binding of oxygen ions to oxygen vacancies [28]. Therefore, we designed bilayer dielectric devices with different Gibbs free energies to improve the performance homogeneity of the devices. In this work, we fabricated and investigated Au/HfO_2_/Pt (S1), Au/Ta_2_O_5_/Pt (S2), and Au/Ta_2_O_5_/HfO_2_/Ta/Pt (S3) devices. Compared with single functional layer devices, S3 devices have enhanced stability, lower switching voltages, and more linear regulation of multiple states. The film roughness was characterized using atomic force microscopy. Importantly, we provide a detailed mechanistic explanation of the S3’s superior performance and ultimately validate the device’s microscopic performance.

## 2. Experiments

Pt/Ti/SiO_2_/Si (Pt) substrate was carefully cleaned with acetone, ethanol, deionized water, and ethanol in an ultrasonic bath, respectively, each for 10 min. Before deposition, the chamber pressure was adjusted to 0.7 Pa. The pressure was maintained using a combination of argon (Ar) and oxygen (O_2_) gases at a total flow rate of 30 sccm. Firstly, the Ta layer was deposited on Pt substrate using radio frequency (RF) magnetron sputtering with a Ta metal target in a pure argon atmosphere (Ar: 30 sccm); the sputtering power was 100 W, and the deposition time was 120 s at RT. Secondly, the HfO_2_ layer was deposited by RF magnetron sputtering with a 99.999% pure HfO_2_ ceramic target; sputtering was carried out at 300 °C for 3 nm in an argon–oxygen (Ar/O_2_ = 15/15 sccm) mixed gas atmosphere. Thirdly, the Ta_2_O_5_ layer was deposited using a 99.999% pure Ta_2_O_5_ ceramic target in an atmosphere of 300 °C with a flow rate of 15 sccm for both argon and oxygen at a power of 60 W for 9 nm. Then, a ~40 nm thick Au top electrode (TE) was deposited onto the thin film by RF sputtering at RT via patterning with a circular shadow mask (*ϕ* = 100 µm). Finally, devices with three structures of S1, S2, and S3 were prepared.

In this work, cross array devices were prepared using photolithography and a double-layer photoresist lift-off process. Cross-electrode strips with a width of 2 μm and a pitch of 10 μm were formed on the Pt substrate. The bottom electrode was patterned by UV lithography using a lithography system, and the 10 nm Ti adhesion layer and the 20 nm Au layer were e-beam evaporated using a Denton e-beam evaporator. After lift-off, the growth processes for the HfO_2_ and Ta_2_O_5_ dielectric layers were the same as above. Finally, the top electrode consisting of 5 nm Ta and 40 nm Au was patterned and deposited using photolithography, e-beam evaporation, and similar lift-off.

All electrical measurements were performed on a Keithley 4200 Semiconductor Parameter Analyzer (KEITHLEY, Cleveland, OH, USA). AFM height images were obtained using a Veeco Multimode AFM microscope in tapping mode (Solver P47-PRO, NT-MDT Co., Moscow, Russia).

## 3. Results and Discussion

**Figure 1:**
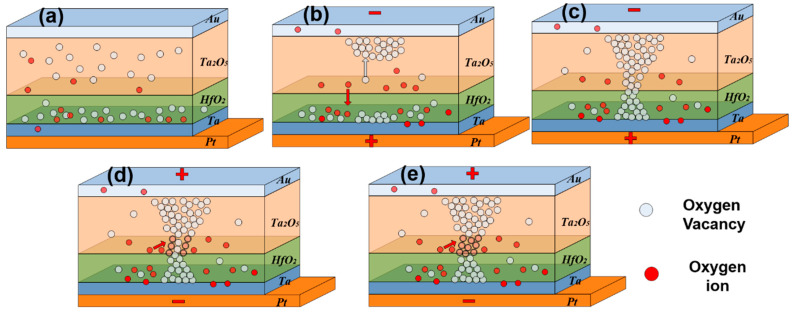
A schematic qualitative model of switching modes in the Au/Ta_2_O_5_/HfO_2_/Ta/Pt device. (**a**) Initial state, (**b**) set process, (**c**) LRS, (**d**) reset process, (**e**) HRS.

We designed the memory resistor device of this work based on the differences in the oxide Gibbs free energy transitions. As shown in Figure 1, we use the resistive transfer mechanism to determine the reasons for the superior performance of Au/Ta_2_O_5_/HfO_2_/Ta/Pt devices. The initial state of the device is shown in Figure 1a, where more oxygen vacancies exist in the hafnium oxide layer near the tantalum side because tantalum is more capable of absorbing oxygen than the tantalum–oxygen interface [29]. The oxygen vacancy content of the hafnium oxide layer was characterized as shown in Figure S1, with an oxygen vacancy content of 42.23%. As shown in Figure 1b, when a negative bias is applied on the top electrode, oxygen in the dielectric layer will undergo the reaction in Equation (1), producing oxygen vacancies and oxygen ions, which migrate toward the bottom electrode, and oxygen vacancies move toward the top electrode under the action of the electric field [30]. The device completes the setup process when oxygen vacancies are connected to the top and bottom electrodes, as shown in Figure 1c.

(1) $$O+2e^{-}=V_{o}^{••}+O^{2-}$$

The lower Gibbs free energy means that the oxidation process is more likely to occur [31]. The magnitude of the Gibbs free energy transitions for oxide formation in Ta_2_O_5_ and HfO_2_ are −1903.2 kJ/mol and −1010.8 kJ/mol, respectively [32,33]. Hence, oxygen ions are more likely to recombine with oxygen vacancies in the tantalum oxide layer. Furthermore, the migration activation energy of oxygen ions at the interface is lower than that of the bulk phase [28]. As a consequence, oxygen ions at the interface between HfO_2_ and Ta_2_O_5_ are more likely to migrate under the proper electric field strength. As shown in Figure 1d and e, when a positive bias voltage is applied to the top electrode of the device, the oxygen ions at the interface migrate and react with the oxygen vacancies in the tantalum oxide layer in a complex reaction, as shown in Equation (2), and a reset process occurs, resulting in the formation of the HRS [30].

(2) $$O^{2-}+V_{o}^{••}=O+2e^{-}$$

Therefore, the connection and breaking of the conductive channel of the device occur at the Ta_2_O_5_/HfO_2_ interface, which results in a more regular change in the conductive path and thus a more uniform distribution of high and low resistance values and operating voltages of the device.

Oxygen ion migration at the Ta_2_O_5_/HfO_2_ interface of S3 devices requires only a smaller voltage to drive compared with single-layer functional layer devices, resulting in a smaller switching voltage. The lower operating voltage results in less heat build-up during the reset process [33], which makes the multi-state regulation of S3 devices more linear.

**Figure 2:**
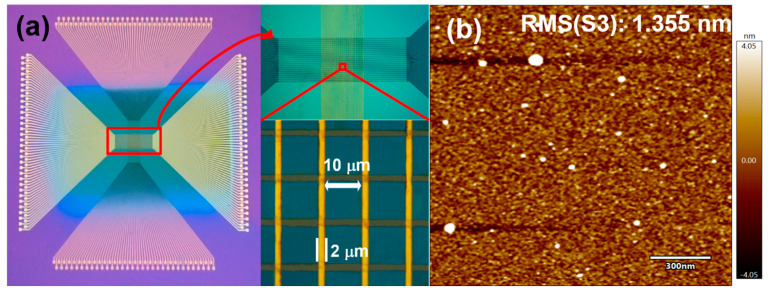
(**a**) Optical image and scanning electron image of the 64 × 64 crossbar array, (**b**) AFM image of S3.

Figure 2a shows that we fabricated a 64 × 64 crossbar array using photolithography and lift-off processes. More details of the crossbar array are shown under the 5× optical microscope image in the upper right corner of Figure 2a. The line width of the array is 2 μm and the spacing is 10 μm, as seen in the 100× optical microscope image in the bottom right of Figure 2a. The surface morphologies of the functional layers of S1, S2, and S3 devices were characterized by AFM, as shown in Figure S2, and the surface roughnesses of the functional layers of S1 and S2 devices were 1.052 nm and 1.175 nm, respectively. as shown in Figure 2b, the surface roughness of the S3 device film was 1.355 nm, which indicates that the fabricated films are relatively flat and suitable for the preparation of memristor devices.

From Figure S3, we can see that the electroforming voltage of the S3 device is higher than that of the S1 and S2 devices, which is due to the fact that the bilayer device requires a larger voltage to drive the oxygen vacancies to form a conductive channel during the electroforming process [7].

**Figure 3:**
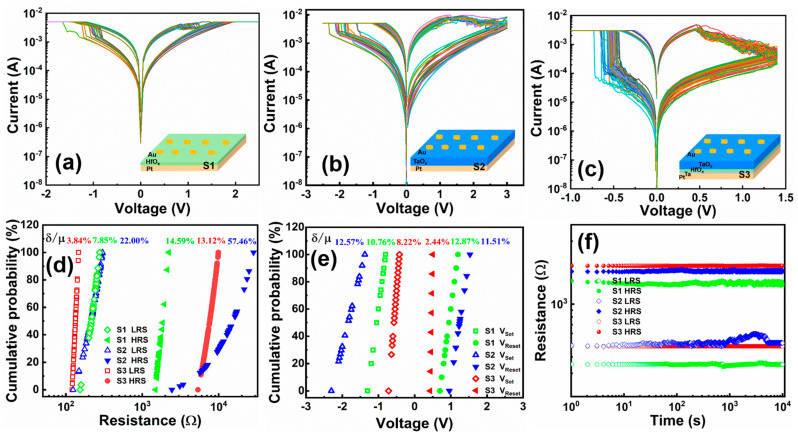
Typical bipolar resistor switch *I*–*V* characteristic curves of S1 (**a**), S2 (**b**), and S3 (**c**). The insets are the corresponding device structures. (**d**) The cumulative probability distribution of the high and low resistance values of the devices. (**e**) Cumulative probability distribution of switching voltages. (**f**) Retention testing of S1, S2, and S3. The relative fluctuations can be expressed by the equation δ/μ (δ is the standard deviation and μ is the mean).

We investigated the S1 and S2 devices. As illustrated in Figure 3a, when a voltage from 0 to −2 V is applied to the S1 device, the SET process occurs at −0.92 V, and the current changes abruptly from 1.5 to 5 mA. When a reverse voltage of 0 to 2.5 V is applied, a RESET process occurs at 0.92 V, and the current fades from 4.3 to 1.4 mA. The S1 device is capable of over 50 DC cycles. The *I*–*V* curve of the S2 device is shown in Figure 3b. When a negative voltage of −2.5 V is applied to the Au electrode, the SET process can be observed at −1.8 V, where the current changes abruptly from 1.8 to 5 mA. When a positive voltage of 3 V is applied, the device switches to the RESET process, and the current changes gradually from 8 mA to 4 mA. The curves were repeated over 70 times. As shown in Figure 3c, by applying a sweep voltage from 0 to −1.0 V to the S3 device, the SET process occurs at −0.54 V and the current suddenly increases from 0.1 to 3 mA. With a reverse positive sweep from 0 to 1.4 V, the device can return to the initial OFF state and the current gradually decreases from 4 to 0.1 mA in one integration cycle. By the same operation method, the S3 device can run steadily for over 200 cycles. This indicates that the S3 device has higher stability than S1 and S2 devices during C2C operation, with significantly lower *V*_Set_ and *V*_Reset_ for S3 compared with S1 and S2, respectively.

Figure 3d shows the cumulative distribution of the high and low resistance values of S1, S2, and S3 at 0.5 V. The switching ratio of the devices is calculated by reading the average of the high and low resistance values of the S1, S2, and S3 devices at 0.5 V. The HRS/ LRS ratio of the S3 device is 58.7, and the ratios of the S1 and S2 devices are 7.2 and 55.2, respectively, which indicates that the S3 device has a larger switching ratio. The results show that the ON/OFF ratio of the S3 device is sufficient for RRAM devices to be used for storing data [24]. Here, relative fluctuations are defined by δ/μ, where δ is the standard deviation and μ is the mean value. The relative HRS volatilities of S1, S2, and S3 devices are 14.59%, 57.46%, and 13.12%, respectively, and the relative LRS volatilities are 7.85%, 22.00%, and 3.84%, respectively. Both the high and low resistance fluctuation coefficients of the S3 device are smaller than those of the S1 and S2 devices, indicating that the S3 device has excellent uniformity. This high degree of homogeneity is due to the different Gibbs free energies of the bilayer devices, as well as the smaller migration energy of oxygen ions at the HfO_2_/Ta_2_O_5_ interface [27,29], which limits the disruption and restoration of the conductive channels to the vicinity of the Ta_2_O_5_ interface where the Gibbs free energies are lower, reduces the randomness of the conductive channel disconnection, and increases the uniformity of the high- and low-resistance states.

Figure 3e shows the cumulative distribution of *V*_Set_ and *V*_Reset_ for S1, S2, and S3. We can see that the δ/μ values of the *V*_set_ of S1, S2, and S3 are 10.76%, 12.57%, and 8.22%, respectively, and the δ/μ values of the *V*_reset_ of S1, S2, and S3 are 12.87%, 11.51%, and 2.44%, respectively. The S3 device has significantly decreased δ/μ compared with the operating voltages corresponding to S1 and S2. Comparative results show that the S3 device is more stable and requires a smaller driving voltage to connect and disrupt the conductive channels of the device, as the oxygen ion mobility energy at the interface is lower than that of the bulk phase. The lower operating voltage assists in reducing power consumption [28].

**Figure 4:**
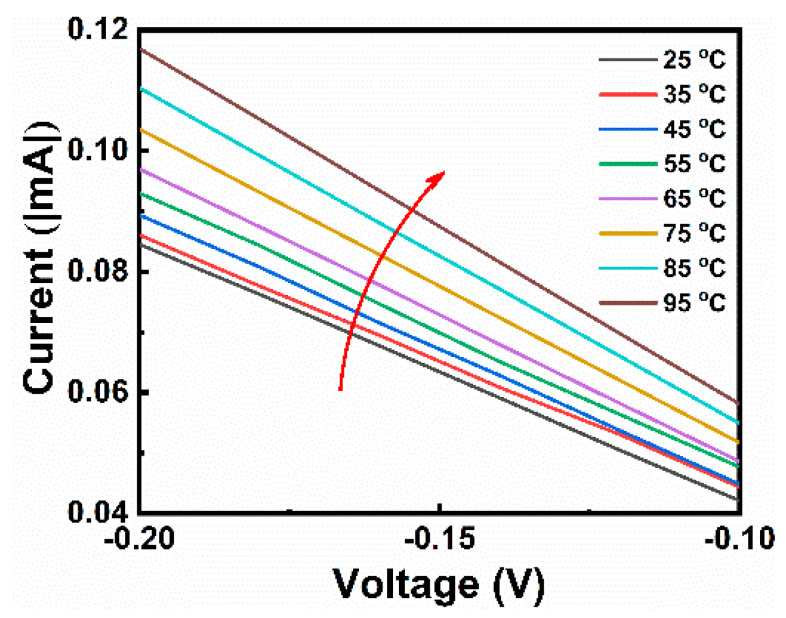
Temperature-dependent *I*–*V* curves for S3 operated in LRS mode at different temperatures, ranging from 25 to 95 °C.

Figure 3f shows the retention performance of the devices. The S1 and S2 devices have good retention performance in both the high- and low-resistance states with very little fluctuation. In comparison, the S3 device has better stability with almost no fluctuation in HRS and LRS over 10^4^ s. As shown in Figure S4, we performed programming endurance tests on S1, S2, and S3 devices. During the voltage pulse fatigue tests, the resistance of S1 and S2 devices changed significantly within 10^5^ pulses, whereas the high and low resistances of S3 devices did not fluctuate significantly within 10^6^ pulses, which indicates that the fatigue resistance of S3 devices is better than that of single-layer devices. These observations suggest that the S3 device has superior storage characteristics. Figure 4 shows the temperature change curve of the S3 device in the low-resistance state, and the on-current increases with temperature, which is consistent with the trend of the oxygen vacancy conductive mechanism. This proves that the conductive channel of the device consists of oxygen vacancies, which is consistent with our proposed conductive mechanism [7].

**Figure 5:**
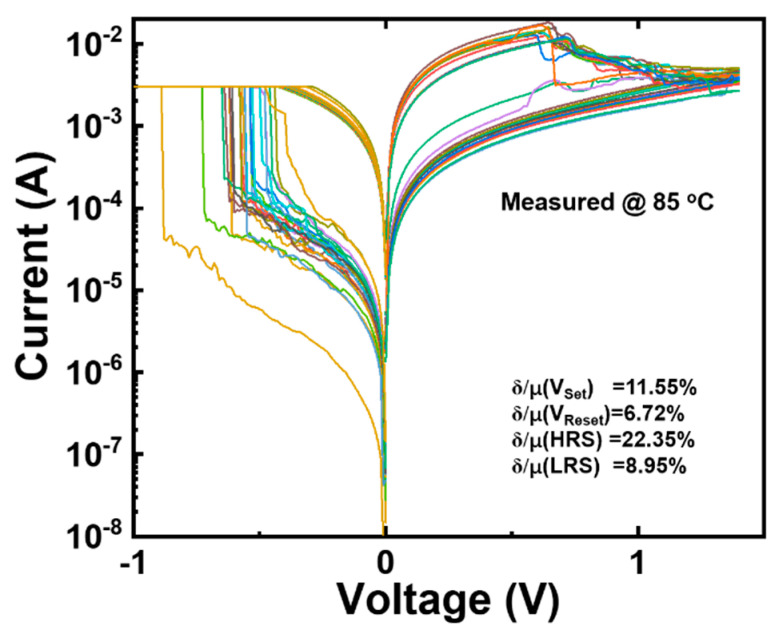
Typical bipolar resistor switch *I*–*V* characteristic curves of the S3 device at 85 °C.

Temperature has a large impact on the performance of the device; therefore, in this work, the *I*–*V* performance of the S3 device was tested in an 85 °C environment, and the results are shown in Figure 5, where the δ/μ values of *V*_Set_, *V*_Reset_, HRS, and LRS are statistically calculated to be 11.55%, 6.72%, 22.35%, and 8.95%, respectively. Compared with the performance of the S3 device at room temperature, the volatility of the test results conducted at 85 °C is increased, which is due to the increase in temperature, which decreases the stability of the oxygen vacancies in the device and leads to an increase in the fluctuations. However, it is clear from the *I*–*V* performance of the devices that the S3 device is still able to function properly in an 85 °C environment.

**Figure 6:**
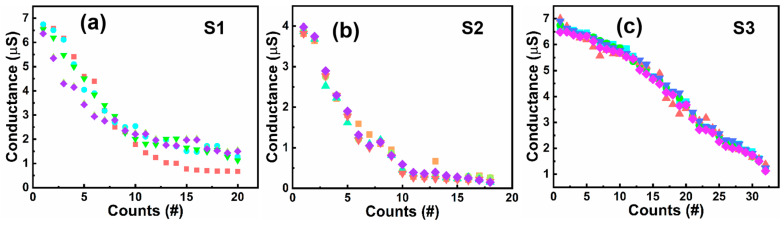
Quantum conductance statistics of five points for (**a**) S1, (**b**) S2, and (**c**) S3 devices, respectively.

Multiple conductance states in memristors have a wide range of applications in areas such as ultrahigh-density information storage, logic storage circuits, and neural networks, and the higher the linearity of the conductance states, the more favorable it is to improve the accuracy of the device in the application [34,35,36]. The polymorphic regulation was obtained by utilizing DC voltage scanning during the device reset process, starting from the voltage at the beginning of the reset and increasing the cut-off voltage in steps of 0.02 V until the end of the reset process. The conductance values obtained from each cut-off voltage regulation were read, and five points were selected for each of the S1, S2, and S3 devices to be modulated. The results of the statistical multistate regulation are shown in Figure 6, where we can see that the S1 and S2 devices have 20 conductance states and 18 conductance states, respectively, adjusted under the control of the cut-off voltage, and the resulting conductance states are slightly less linear. Compared with the S1 and S2 devices, our S3 device can regulate up to 32 conduction states with higher linearity than the S1 and S2 devices. This is because the resetting process of the stacked structure of the S3 device occurs at the interface of hafnium oxide and tantalum oxide, which reduces the randomness of the conductive channel changes and improves the linearity of the multiple conductive states of the S3 device. Finally, the yield of the S3 device in the array shown in Figure 2a was tested, as shown in Figures S5 and S6. The yield of the device reached 79/81 × 100% ≈ 97.5%, and the device-to-device uniformity of the S3 device is 92.37%, which indicates that it has good micro-miniaturization potential.

As summarized in Table 1, in comparison with other literature on the same device structure, the present work has a lower switching voltage and 32 adjustable conductance states, which are important for the optimization of the device performance.

**Figure 7:**
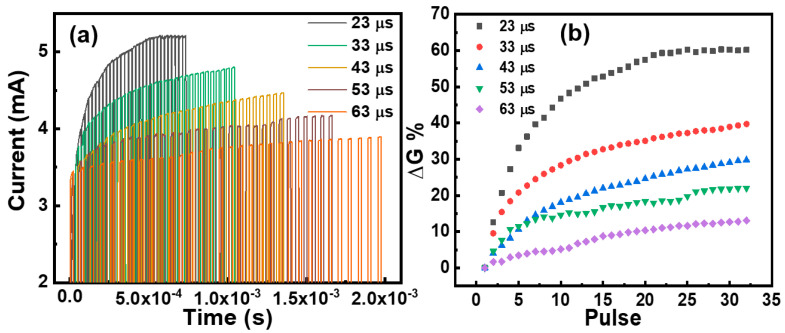
Pulse-frequency dependent characteristics of the device. (**a**) Voltage pulses of different periods regulate the change in the current of the S3 device. (**b**) Variations in the rate of change of conductance with the period of applied pulses.

Due to the good stability of the S3 device, its conductance was regulated using a pulse voltage. As shown in Figure 7a, we applied a pulse voltage with an amplitude of 0.6 V and a pulse width of 3 μs to regulate the conductance of the S3 device by changing the period of the pulse. After applying 32 pulse voltages, the maximum change in current was achieved by pulse regulation with a period of 23 μs, and the minimum change in current was achieved by pulse regulation with a period of 63 μs. The conductance change rate obtained by pulse regulation is shown in Figure 7b. It can be seen that for the same number of pulses, the conductance change rate of the pulse voltage regulation with a pulse period of 23 μs is more than 60%, while the conductance change rate of the pulse voltage regulation with a pulse period of 63 μs is only 10%. A good frequency-dependent property is shown, and this property can be used for frequency-dependent synaptic learning behavior [8,40].

## 4. Conclusions

In conclusion, we prepared double oxide layers with different Gibbs free energies as functional layers and compared them with single functional layer devices. The Au/Ta_2_O_5_/HfO_2_/Ta/Pt devices have a larger switching ratio of 58.7, *V*_Set_ and *V*_Reset_ as low as −0.55 V and 0.46 V, respectively, and operating voltages that are smaller than those of S1 and S2 devices. Analysis of the statistical distributions of the switching voltage and resistance values shows that the δ/μ values of the *V*_Set_, *V*_Reset_, HRS, and LRS are only 8.22%, 2.44%, 13.12%, and 3.84%, respectively, which are smaller than the corresponding relative fluctuations of the single-layer devices. This indicates that the uniformity of the device is improved. The interface effect of the functional layer in the S3 device makes its multi-state modulation more linear. We present a detailed physical mechanism of resistive switching to explain the device’s performance enhancement. High yields were obtained in the verification of the device’s microscale performance. The Au/Ta_2_O_5_/HfO_2_/Ta/Pt RRAM devices proposed in this study show great potential for nonvolatile memory applications, in-store computing, and micro-shrinkage integration and provide a new idea for the design and fabrication of resistor devices with stable performance.

## Figures and Tables

**Table 1:** Comparison of electrical properties with other literature.

Device Structure	*V*_Set_ (V)	*V*_Reset_ (V)	*δ* (*V*_Set_)	*δ* (*V*_Reset_)	On/Off	Multiple Conductivity States	Ref.
Pt/Ta_2_O_5_/HfO_2-*x*_/TiN	15	−10	NA	NA	10^4^	NA	[37]
Pt/Ta_2_O_5_/HfO_2-*x*_/Hf	5.5	−3.5	NA	NA	10^3^	NA	[38]
TiN/HfO_2_/Ta_2_O_5_/Ta	−3	3	NA	NA	10^2^	NA	[39]
TiN/TaO*_x_*/HfO_2_/TiN	1.75	−0.6	NA	NA	13.4	NA	[40]
Au/Ta_2_O_5_/HfO_2_/Ta/Pt	0.5	0.48	8.22%	2.44%	58.7	32	This Work

Note: NA is not available.

## Data Availability

Data are contained within the article and Supplementary Materials.

## Supplementary Materials

The following supporting information can be downloaded at: https://www.mdpi.com/article/10.3390/mi15050605/s1. Figure S1: XPS testing of HfO_2_ thin film. Figure S2: Surface morphology testing of device films:(a) S1, (b) S2. Figure S3: Electroforming process of S1, S2, and S3 devices. Figure S4: Programming endurance tests for devices: (a) S1, (b) S2, (c) S3. Figure S5: Figure 2a DC I-V cycle test of 81 devices in the array. Figure S6: Statistics on the number of *I*–*V* cycles in Figure S5.
