# Supplementary material for: Enhancing the Uniformity of a Memristor Using a Bilayer Dielectric Structure
Source: Micromachines (Basel). 2024 Apr 30;15(5):605. doi: 10.3390/mi15050605 (PMC11123252; doi:10.3390/mi15050605)
Supplement: Supplementary file 1 [file micromachines-15-00605-s001.zip › micromachines-2958282-supplementary.pdf]

Enhancing the Uniformity of a Memristor Using a Bilayer Dielectric Structure

Supplementary experiments

The X-ray photoelectron spectroscopy (XPS) (ESCALAB 250Xi, Thermo Fisher Inc., Waltham, MA, USA) analysis of the samples were performed. Phase identification and crystal structures of the as-deposited flms were performed by Xray diffraction (XRD, D8 Advance, Bruker, German) with Cu Ka radiation.

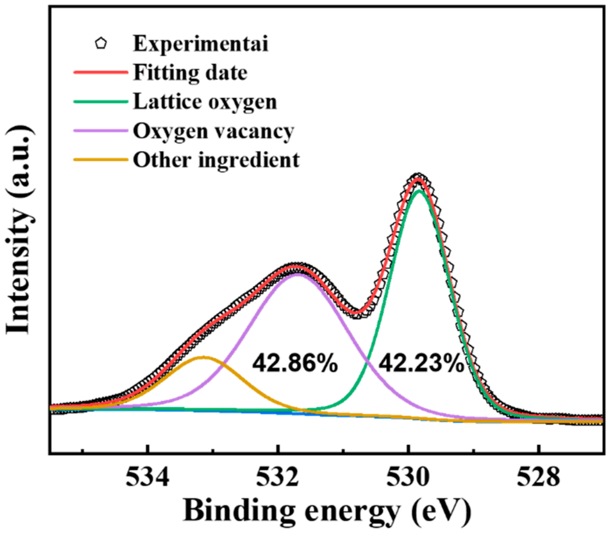

Figure S1. XPS testing of HfO_2 thin film.

From the XPS test of the hafnium oxide dielectric layer we can see that the content of lattice oxygen in the hafnium oxide layer is 42.86% and the content of oxygen vacancies is 42.23%.

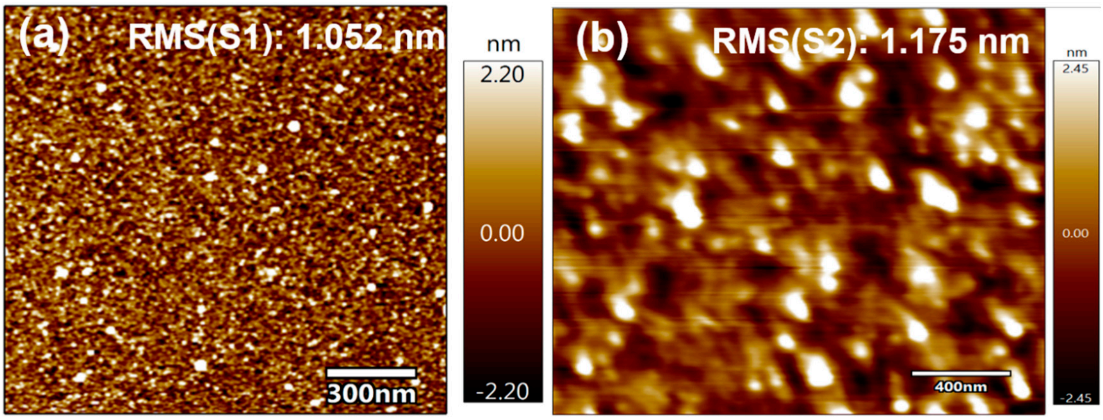

Figure S2. Surface morphology testing of device films: (a) S1, (b) S2.

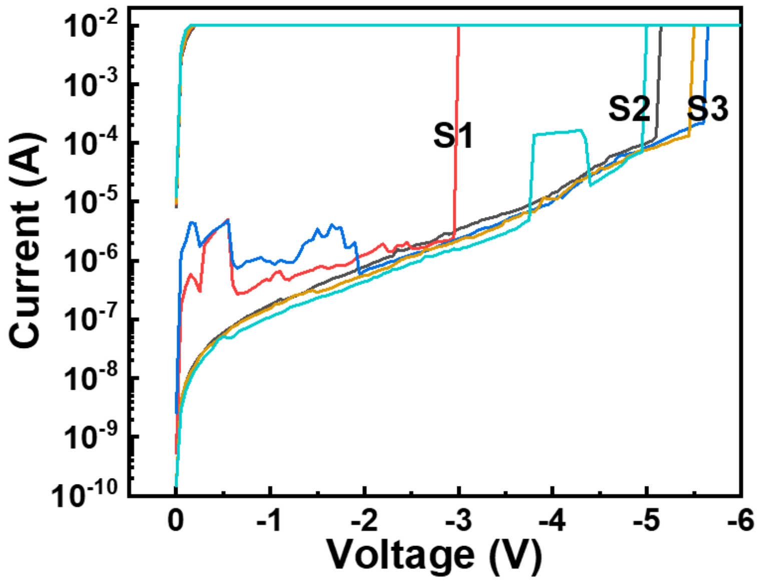

Figure S3. Electroforming process of S1, S2 and S3 devices.

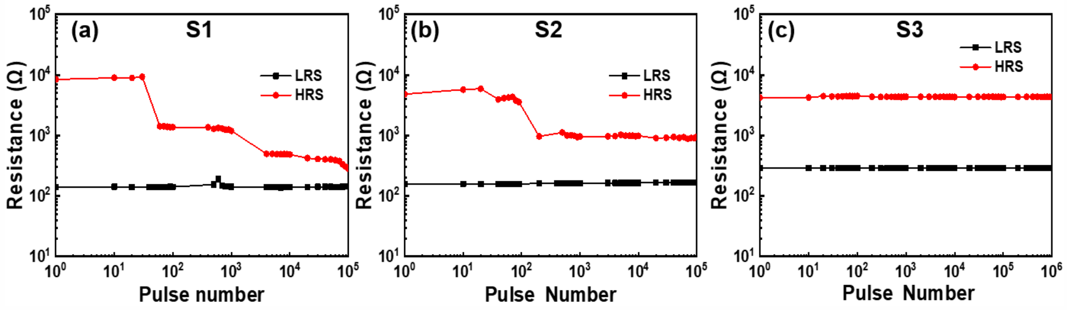

Figure S4. Programming endurance tests for devices: (a) S1, (b) S2, (c) S3.

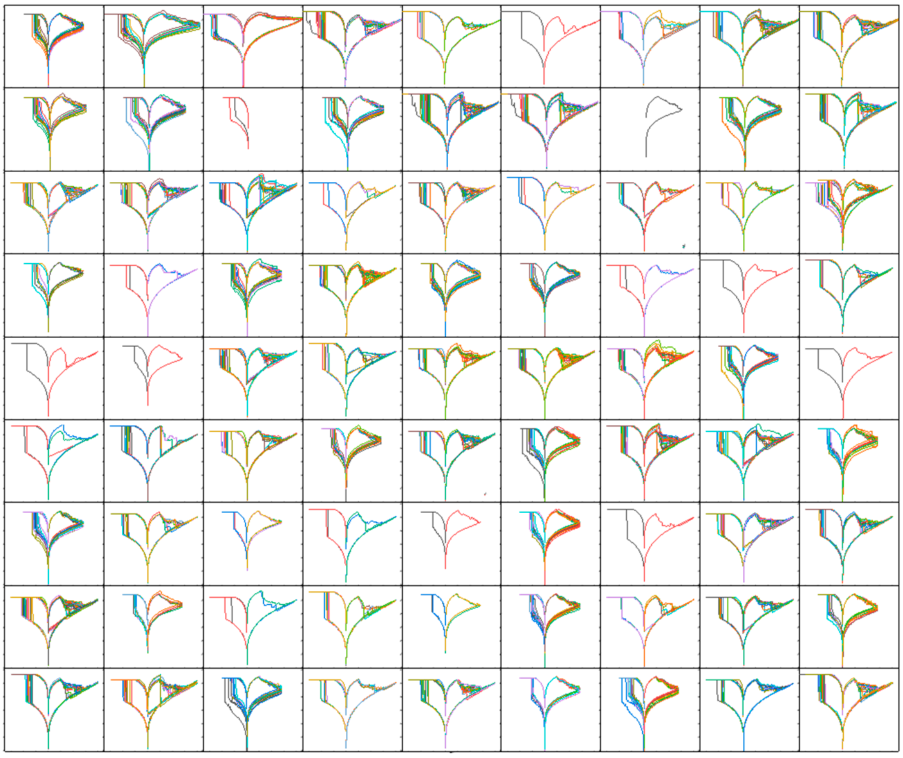

Figure S5. Figure 2a DC *I*-*V* cycle test of 81 devices in the array.

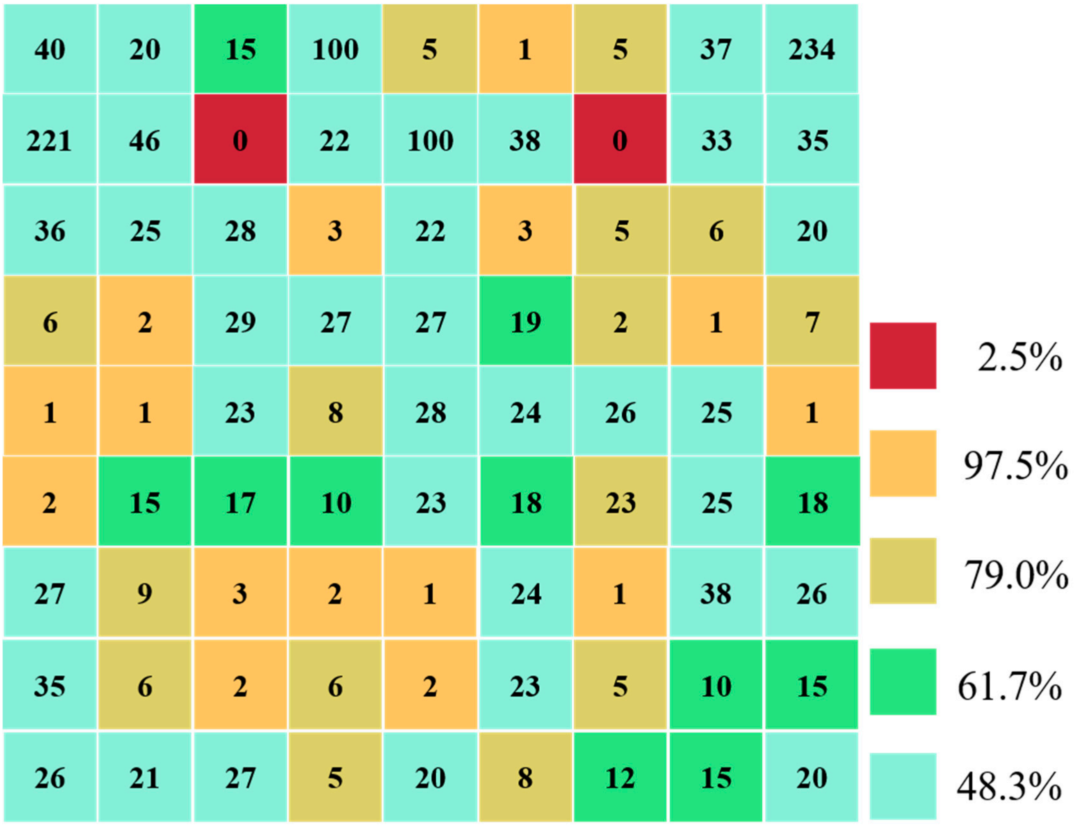

Figure S6. Statistics on the number of *I*-*V* cycles in Figure S5

It can be seen from Figure S6 that the prepared array has a high yield, reaching 97.5%, and the number of devices that can work for more than 5 cycles is 79%.
